# The promoter T-413A variant and elevated enzyme levels of heme oxygenase-1 associated with an increased risk of polycystic ovarian syndrome

**DOI:** 10.3389/fendo.2025.1644373

**Published:** 2025-11-04

**Authors:** Qiuyi Wang, Jiagui Liang, Qingqing Liu, Hongwei Liu, Huai Bai, Wei Huang, Ping Fan

**Affiliations:** 1 Department of Obstetrics and Gynecology, West China Second University Hospital, Sichuan University, Chengdu, Sichuan, China; 2 Laboratory of Genetic Disease and Perinatal Medicine, Key Laboratory of Birth Defects and Related Diseases of Women and Children, Ministry of Education, West China Second University Hospital, Sichuan University, Chengdu, Sichuan, China; 3 Department of Reproductive Medical Center, West China Second University Hospital, Sichuan University, Chengdu, Sichuan, China

**Keywords:** heme oxygenase-1, genetic polymorphism, polycystic ovarian syndrome, oxidative stress, metabolism

## Abstract

**Background:**

Oxidative stress and metabolic disorders significantly contribute to the development of polycystic ovarian syndrome (PCOS). Heme oxygenase-1 (HMOX1) plays a key role in the degradation of heme and the regulation of oxidative stress, ferroptosis, and glycolipid metabolism. This study explored the relationship between *HMOX1* promoter T-413A single nucleotide polymorphism (SNP, rs2071746), (GT)n dinucleotide repeat variant (rs3074372), plasma HMOX1 levels, and the risk of PCOS in Chinese women.

**Methods:**

This case-control study included 1092 women diagnosed with PCOS and 805 controls. The (GT)n and rs2071746 polymorphisms were identified using polymerase chain reaction amplification, followed by capillary electrophoresis or restriction fragment length polymorphism. HMOX1 levels and clinical, metabolic, hormonal, and oxidative stress indices were analyzed.

**Results:**

The *HOMX1* rs2071746T/A SNP was associated with an increased risk of PCOS based on genotype, recessive, dominant, and allele genetic models (*P* < 0.05). After adjusting for age, body mass index, and recruitment year of participants, the dominant model (odds ratio [OR] = 1.272, 95% confidence interval [CI]: 1.013–1.597, *P* = 0.039) and the TT genotype (OR = 1.395, 95% CI: 1.033–1.883, *P* = 0.030, with the AA genotype as the reference) remained a significant predictor of PCOS in the logistic regression models. No significant differences were observed in the (GT)n polymorphism of *HMOX1* based on different genetic models. However, the TT/SS combined genotype of *HMOX1* rs2071746T/A and (GT)n polymorphisms was associated with an increased risk of PCOS (OR = 1.442, 95% CI: 1.021–2.035, *P* = 0.037). Furthermore, elevated HMOX1 levels were related to a slight but significant increase in the risk of PCOS, and the rs2071746T/A and (GT)n genetic variants significantly affected obesity, oxidative stress, endocrine abnormalities, and metabolic disorders.

**Conclusion:**

*HMOX1* rs2071746T/A variant and elevated plasma HMOX1 levels are associated with an increased risk of PCOS.

## 1 Introduction

Polycystic ovarian syndrome (PCOS) is the leading endocrine-metabolic disorder among women of reproductive age, with a prevalence of 10–13% (1). Its clinical presentation is diverse and may include reproductive, dermatological, metabolic, and psychological symptoms such as irregular menstrual cycles, infertility, acne, hirsutism, obesity, insulin resistance, dyslipidemia, anxiety, depression, etc. (1, 2). PCOS can affect women throughout their lives and potentially lead to long-term complications, including type 2 diabetes (T2D), cardiocerebrovascular disease, and endometrial cancer. The exact etiology of PCOS remains unknown, making its treatment difficult (1, 3). Increasing evidence indicates that its etiology is complex, involving interactions between multiple predisposing genes, genetic epigenetics, and detrimental environmental factors (2, 4–6).

Heme oxygenase (HMOX) is the rate-limiting enzyme that catalyzes the breakdown of heme, resulting in the production of biliverdin (BV), carbon monoxide (CO), and free iron (Fe^2+^) (7, 8). BV is rapidly turned into bilirubin (BR) by BV reductase (8, 9). Heme and free Fe^2+^ are toxic owing to their oxidative properties; however, in the physiological state, free iron is swiftly sequestered by ferritin, ensuring a harmonious balance within the body (8). CO, BV, and BR possess vasodilator, antioxidant, and anti-inflammatory properties, but excessive accumulation of these products can be toxic (8, 10). The two main isomers of HMOX in humans, inducible HMOX1 (also known as HO-1) and constitutive HMOX2 (also known as HO-2), have similar structures and catalytic functions but are distributed in different tissues and exhibit distinct features (10). Low HMOX1 expression was observed in most tissues under normal conditions. However, its expression can be markedly increased in response to different pathophysiological stress conditions or stimulation factors (9, 11).

The *HMOX1* is located on chromosome 22q13.1. Two genetic polymorphisms in the promoter of *HMOX1*, the (GT)n dinucleotide repeat variant (rs3074372) and rs2071746T/A single nucleotide polymorphism (SNP) (rs2071746), can affect the transcriptional activity of *HMOX1* (11). These two polymorphisms are closely linked to certain diseases, including sensitivity to several cancers and coronary heart disease (11), cardiovascular events and mortality in patients undergoing hemodialysis (12), T2D (13), pre-eclampsia (14), chronic obstructive pulmonary disease (15), SARS-CoV-2 viremia in COVID-19 infection (16), and risk of encephalitis in HIV infection (17).

Oxidative stress and metabolic disorders significantly contribute to the pathophysiology and progression of PCOS (1, 18–21). Genetic variants of *HMOX2* G554A and A-42G SNPs are associated with endocrine abnormalities and glycolipid metabolic irregularities in patients with PCOS (5). The levels of *HMOX1* mRNA are higher in subcutaneous adipose tissue and granulosa cells (22, 23), but the concentrations of serum HMOX1 are lower in women with PCOS than those among control women (24). However, the association between *HMOX1* polymorphisms and PCOS remains unclear. Therefore, we explored the relationship between *HMOX1* (GT)n repeats and rs2071746T/A polymorphisms and the risk of PCOS. Additionally, we analyzed how these genetic variants affected plasma HMOX1 levels and various clinical and biochemical parameters in Southwest Chinese women.

## 2 Materials and methods

### 2.1 Study participants

**Figure 1:**
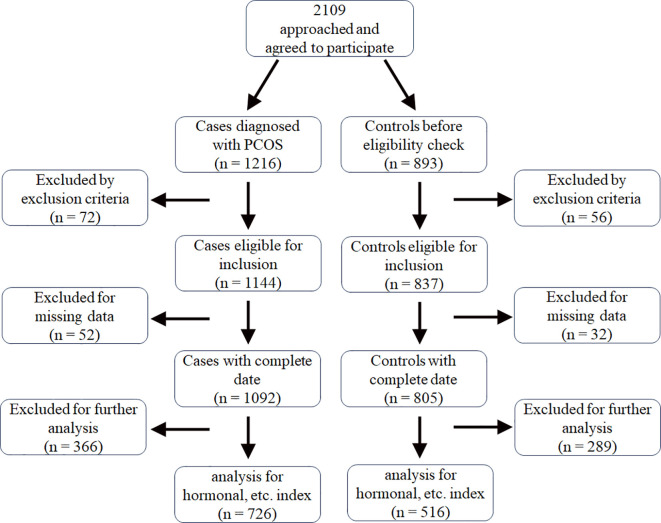
The process of recruitment and selction of the case and control group.

This was a case-control study. All participants aged 17–40 years provided written informed consent and were recruited from the Reproductive Endocrinology Outpatient Department of the West China Second University Hospital between 2006 and 2024 (**Figure 1**). This study was approved by the Institutional Review Board of West China Second University Hospital, Sichuan University (2014–014 for P. Fan).

PCOS was diagnosed based on the revised 2003 Rotterdam European Society of Human Reproduction and Embryology/American Society for Reproductive Medicine consensus criteria, which include irregular menstrual cycles, oligo-ovulation, or anovulation (OA), biochemical and/or clinical hyperandrogenism (HA), and polycystic ovaries (PCOs) (25). Detailed definitions of HA, OA, and PCOs have been provided in previous articles (26–28). PCOS was diagnosed if a woman met at least two of the three criteria after ruling out other etiologies such as Cushing syndrome, congenital adrenal hyperplasia, and androgen-secreting tumors (1, 25). Both HA and OA are required in individuals aged < 20 years of age (1). Control women had regular menstrual cycles between 21–35 days, normal ovarian morphology on ultrasonography, and no biochemical or clinical signs of HA.

The participants were excluded if they had infections, cardiovascular diseases, liver or kidney diseases, autoimmune diseases, thyroid disorders, hypogonadism, hyperprolactinemia, premature ovarian insufficiency, endometriosis, or other malignancies. Additionally, the participants were excluded from the analysis when comparing oxidative stress and metabolic and hormonal parameters between groups/subgroups if they met any of the following criteria: (i) use of hormonal therapy and/or medications that influence glucose and lipid metabolism within 12 weeks before the study; (ii) smoking; (iii) being pregnant or in the luteal phase of their menstrual cycle; and/or (iv) having a fasting glucose (Glu) level of ≥7.0 mmol/L and/or a 2-hour plasma glucose after the glucose challenge (2-h Glu) level of ≥11.1 mmol/L in the control group.

Clinical indices, including waist circumference (WC), body mass index (BMI), waist-to-hip ratio, diastolic blood pressure (DBP), systolic blood pressure, severity of acne and hirsutism, and ovarian volume were assessed as previously described (27–29).

Blood samples were collected after fasting for 8–12 h. Blood cells were stored at 4°C, and plasma and serum aliquots were preserved at −80°C for later analysis. A 75g oral glucose tolerance test was conducted immediately after fasting blood sampling.

### 2.2 DNA purification and genotype measurements

Genomic DNA was purified from stored blood cells using a previously described method (30). (GT)n repeats in *HMOX1* were determined using polymerase chain reaction (PCR) amplification and capillary electrophoresis. The forward primer with 6-carboxyfluorescein (FAM) was 5′-FAM-CCAGCTTTCTGGAACCTTCTG-3′, the reverse primer was 5′-GAAACAAAGTCTGGCCATAGGA-3′ (31). Samples were amplified using a touchdown PCR protocol (17). The products were then analyzed using a 3730xl DNA Analyzer (Applied Biosystems, Foster City, CA, USA) and GeneMapper 4.1 (Applied). Short repeats, with fewer than 27 GT repeats, were classified as S alleles, whereas long repeats, with at least 27 GT repeats, were classified as L alleles (12). For genotyping the rs2071746 SNP, we used PCR and restriction fragment length polymorphism method with a mismatched primer set (the forward primer: 5′-GTTCCTGATGTTGCCCACCAAGC-3′; the reverse primer: 5′-CTGCAGGCTCTGGGTGTGATTTTG-3′). The PCR products of the rs2071746T/A SNP (151 bp) were then digested with *HindIII* (New England Biolabs, Ipswich, MA, USA), resulting in 20 and 131 bp fragments with the T allele and a whole 151-bp product with the A allele. The results were verified by repeating the genotyping of > 30% of the randomly selected samples, yielding 100% concordance.

### 2.3 Analysis of HMOX1, oxidative stress, hormonal, and metabolic indices

Plasma HMOX1 concentrations were measured using ELISA kits (Elabscience Biotechnology Co., Ltd., Wuhan, China).

Estradiol, TT, luteinizing hormone (LH), follicle-stimulating hormone (FSH), sex hormone binding globulin (SHBG), plasma insulin (Ins) and Glu levels, triglycerides (TG), total cholesterol (TC), high-density lipoprotein cholesterol (HDL-C), low-density lipoprotein cholesterol (LDL-C), apolipoprotein (apo) A1, and apoB concentrations, total antioxidant capacity (T-AOC), glutathione (GSH), total oxidant status (TOS), and oxidative stress index (OSI) were also analyzed as previously described in other studies (20, 21, 28). The homeostatic model assessment of insulin resistance (HOMA-IR) and free androgen index (FAI) were calculated as previously described (18, 28) using the following formulas:


HOMA−IR = fasting Glu (mmol/L) × fasting Ins (μU/mL)/22.5

FAI = TT (nmol/L)/SHBG (nmol/L) × 100

### 2.4 Statistical analysis

Data are presented as mean ± standard deviation. We used analysis of variance or independent sample t-tests for normally distributed variables and the Mann–Whitney U test for non-normally distributed variables. Analysis of covariance was used to evaluate differences in clinical and biochemical indices after adjusting for variations in age, BMI, and participant recruitment year. Chi-squared (χ²) analysis was performed to evaluate deviations in genotypic distribution from Hardy–Weinberg equilibrium and to compare the frequencies of genotypes and alleles between two groups. The Spearman’s correlation coefficient was used to assess the relationship between HMOX1 levels and other parameters. Differences were considered statistically significant if the *P* value was <0.05. Data were analyzed using the Statistical Program for Social Sciences (SPSS) 21.0 (IBM SPSS Statistics, IBM Corporation).

Power values were calculated according to the disease allele frequency of the rs2071746T/A SNP in *HMOX1* and the sample size (prevalence = 0.12, significance level = 0.05) using the Genetic Association Study Power Calculator (http://csg.sph.umich.edu/abecasis/gas_power_calculator/index.html).

Using the online SNPStats (https://www.snpstats.net/start.htm), we assessed the linkage disequilibrium between two genetic polymorphic loci in view of the D’ parameter.

## 3 Results

### 3.1 Clinical and biochemical characteristics of the participants

Owing to the significant discrepancies in BMI and age between the PCOS and control groups (**Table 1**), and the relatively long recruitment period of participants between 2006 and 2024 in this study, we adjusted for these three confounding factors in our subsequent analyses.

**Table 1:** Clinical and biochemical parameters in women with PCOS and controls.

	Controls (n = 805)	PCOS (n = 1092)	*P*	*P^a^*
Age (years)	28.25 ± 4.10	25.10 ± 4.15	<0.001	
BMI (kg/m^2^)	21.15 ± 2.81	23.04 ± 4.14	<0.001	
WC (cm)	73.64 ± 8.08	79.15 ± 11.07	<0.001	<0.001
Waist-to-hip ratio	0.81 ± 0.60	0.85 ± 0.07	<0.001	<0.001
F-G score	0.24 ± 0.72	1.74 ± 2.03	<0.001	<0.001
Acne grade score	0.13 ± 0.34	0.67 ± 0.90	<0.001	<0.001
SBP (mmHg)	112.38 ± 11.15	114.16 ± 10.61	0.001	0.315
DBP (mmHg)	73.33 ± 8.58	75.43 ± 8.75	<0.001	0.003
Mean ovarian volume (mL)	7.36 ± 2.90	9.92 ± 4.04	<0.001	<0.001
Hormonal levels*				
E_2_ (pmol/L)	313.87 ± 324.44	275.17 ± 273.34	0.035	0.657
TT (nmol/L)	1.45 ± 0.52	2.26 ± 0.80	<0.001	<0.001
SHBG (nmol/L)	55.32 ± 27.19	32.63 ± 19.03	<0.001	<0.001
FAI	3.14 ± 1.89	9.53 ± 6.87	<0.001	<0.001
LH (IU/L)	7.05 ± 6.24	13.15 ± 8.05	<0.001	<0.001
FSH (IU/L)	6.55 ± 2.63	6.05 ± 2.10	0.001	0.015
LH/FSH	1.16 ± 1.13	2.24 ± 1.23	<0.001	<0.001
Metabolic profile*				
Fasting Ins (pmol/L)	60.37 ± 35.46	98.46 ± 72.40	<0.001	<0.001
2-h Ins (pmol/L)	365.80 ± 270.73	715.01 ± 566.96	<0.001	<0.001
Fasting Glu (mmol/L)	5.23 ± 0.47	5.34 ± 0.84	0.003	0.861
2-h Glu (mmol/L)	5.98 ± 1.27	7.21 ± 2.46	<0.001	<0.001
HOMA-IR	2.21 ± 1.30	3.79 ± 3.01	<0.001	<0.001
TG (mmol/L)	1.00 ± 0.54	1.41 ± 1.14	<0.001	<0.001
TC (mmol/L)	4.25 ± 0.72	4.43 ± 0.80	<0.001	<0.001
HDL-C (mmol/L)	1.51 ± 0.33	1.37 ± 0.34	<0.001	0.002
LDL-C (mmol/L)	2.35 ± 0.64	2.60 ± 0.77	<0.001	<0.001
TG/HDL-C	0.73 ± 0.61	1.19 ± 1.37	<0.001	<0.001
ApoA1 (g/L)	1.46 ± 0.21	1.42 ± 0.21	0.001	0.752
ApoB (g/L)	0.75 ± 0.17	0.83 ± 0.20	<0.001	<0.001
Oxidative stress parameters*				
TOS (nmol H_2_O_2_ Equiv./mL)	11.41 ± 5.34	15.25 ± 10.31	<0.001	<0.001
T-AOC (U/mL/min)	14.51 ± 2.60	15.92 ± 3.51	<0.001	<0.001
OSI	0.79 ± 0.41	0.99 ± 0.76	<0.001	<0.001
GSH (nmol/mL)	1.11 ± 0.25	1.18 ± 0.25	<0.001	0.005
TOS/GSH	10.50 ± 5.76	12.71 ± 9.33	<0.001	<0.001
HMOX1 (μg/L)	4.51 ± 2.41	5.02 ± 4.61	0.018	0.011

Values are presented as average ± standard deviation.

apoA1, apolipoprotein A1; apoB, apolipoprotein B; BMI, body mass index; DBP, diastolic blood pressure; E_2_, estradiol; FAI, free androgen index; F-G score, Ferriman–Gallwey score; FSH, follicle-stimulating hormone; Glu, glucose; GSH, glutathione; HDL-C, high-density lipoprotein cholesterol; HMOX1, heme oxygenase-1; HOMA-IR, the homeostatic model assessment of insulin resistance; Ins, insulin; LDL-C, low-density lipoprotein cholesterol; LH, luteinizing hormone; OSI, oxidative stress index; SBP, systolic blood pressure; SHBG, sex hormone-binding globulin; T-AOC, total antioxidant capacity; TC, total cholesterol; TG, triglycerides; TOS, total oxidant status; TT, total testosterone; WC, waist circumference. 2-h Ins and 2-h Glu, 2-hour plasma insulin and glucose after the glucose challenge.

*P* Continuous variables were compared between the two groups using the independent samples t-test (normally distributed) or the Mann-Whitney U test (non-normally distributed).

*P^a^* Comparisons of the parameters were corrected for differences in age, BMI, and recruitment year of participants between the two groups using analysis of covariance.

*Controls (n = 516), PCOS (n = 726).

**Table 1** shows that the PCOS group had significantly higher acne grade scores, F-G scores, average ovarian volumes, DBP, WC, waist-to-hip ratio, TT, LH, LH/FSH ratio, FAI, fasting Ins, HOMA-IR, 2-h Glu and 2-hour insulin after the glucose challenge (2-h Ins), LDL-C, TC, TG, TG/HDL-C ratio, apoB, TOS, T-AOC, GSH, OSI, TOS/GSH ratio, and plasma HMOX1 levels, but lower serum FSH, SHBG, and HDL-C concentrations than the control group (*P* < 0.05).

We further compared the plasma HMOX1 levels in lean (BMI < 23 kg/m²) and overweight/obese (BMI ≥ 23 kg/m² and/or waist circumference > 80 cm) subgroups after adjusting for age, BMI, and recruitment period of participants. The results showed that the HMOX1 levels were higher in the lean PCOS subgroup (n = 339) than in the lean control subgroup (n = 382) (5.00 ± 5.00 *vs.* 4.48 ± 2.36 µg/L, *P* = 0.034), but no statistical significance in the overweight/obese PCOS subgroup (n = 387) than in the overweight/obese control subgroup (n = 134) (5.04 ± 4.29 *vs.* 4.48 ± 2.15 µg/L, *P* = 0.115).

### 3.2 Correlation of HMOX1 levels with clinical and biochemical indicators and risk of PCOS

The Spearman’s correlation analysis showed that plasma HMOX1 levels were positively correlated with 2-h Glu, WC, fasting Ins, HOMA-IR, TG/HDL-C ratio, BMI, 2-h Ins, TC, fasting Glu, FAI, T-AOC, apoB, and WHR in patients with PCOS (r = 0.138, 0.132, 0.132, 0.129, 0.127, 0.118, 0.105, 0.093, 0.092, 0.087, 0.085, 0.080, and 0.079, respectively; *P* < 0.05). Although statistically significant, the correlations between HMOX1 and PCOS traits were quantitatively modest.

Binary logistic regression analysis demonstrated that elevated HMOX1 levels were associated with an increased risk of PCOS after correcting for differences in participant recruitment year, age, and BMI (odds ratio [OR] = 1.053, 95% confidence interval [CI]: 1.008–1.100, *P* = 0.019).

### 3.3 Distributions of *HOMX1* rs2071746T/A and (GT)n genotypes and alleles

**Table 2** summarizes the genetic models for the rs2071746T/A and (GT)n repeat polymorphisms in *HOMX1*. The distribution of genotypes for both polymorphisms was consistent with Hardy–Weinberg equilibrium in women with and without PCOS (*P* > 0.05).

**Table 2:** Association of *HMOX1* T-413A (rs2071746) and (GT)n repeat polymorphisms with the risk of PCOS using different genetic models.

Variants	Controls (n = 805)	PCOS (n = 1092)	Unadjusted		Adjusted	
			OR (95% CI)	*P*	OR (95% CI)	*P*
T-413A (rs2071746)						
Genotype						
AA	173 (21.5%)	193 (17.7%)	Referent			
AT	426 (52.9%)	556 (50.9%)	1.170 (0.919–1.489)	0.201	1.140 (0.872–1.493)	0.342
TT	206 (25.6%)	343 (31.4%)	1.493 (1.141–1.952)	0.003	1.395 (1.033–1.883)	0.030
*P*_HWE_	0.233	0.456				
Recessive						
TT + AT	632 (78.5%)	899 (82.3%)				
AA	173 (21.5%)	193 (17.7%)	1.275 (1.014–1.603)	0.037	1.212 (0.936–1.570)	0.144
Dominant						
TT	206 (25.6%)	343 (31.4%)				
AA + AT	599 (74.4%)	749 (68.6%)	1.332 (1.086–1.632)	0.006	1.272 (1.013–1.597)	0.039
Allele						
A	772 (48.0%)	942 (43.1%)				
T	838 (52.0%)	1242 (56.9%)	1.215 (1.067–1.382)	0.003	/	/
(GT)n repeat						
Genotype						
LL	224 (27.8%)	298 (27.3%)	Referent			
SL	422 (52.4%)	542 (49.6%)	0.965 (0.779–1.197)	0.748	0.935 (0.736–1.189)	0.584
SS	159 (19.8%)	252 (23.1%)	1.191 (0.915–1.551)	0.193	1.134 (0.844–1.522)	0.404
*P*_HWE_	0.292	0.983				
Recessive						
LL + SL	646 (80.2%)	840 (76.9%)				
SS	159 (19.8%)	252 (23.1%)	1.219 (0.975–1.524)	0.082	0.842 (0.655–1.081)	0.178
Dominant						
LL	224 (27.8%)	298 (27.3%)				
SS + SL	581 (72.2%)	794 (72.7%)	0.973 (0.794–1.193)	0.796	1.041 (0.827–1.309)	0.733
Allele						
L	870 (54.0%)	1138 (52.1%)				
S	740 (46.0%)	1046 (47.9%)	1.081 (0.950–1.229)	0.239	/	/

The frequencies of the TT genotype and T allele in the *HOMX1* rs2071746T/A SNP were significantly higher in the PCOS group than those in the control group. The OR indicated that this difference was statistically significant for the dominant model, the recessive model, and the TT vs. AA genotype model, and the allele model (all *P* < 0.05). After adjusting for age, BMI, and recruitment year of participants, the dominant genetic model remained statistically significant in the binary logistic regression model (OR = 1.272, 95% CI: 1.013–1.597, *P* = 0.039) and the TT genotype remained a significant predictor for PCOS in a multinomial logistic regression model, with the AA genotype as the reference (OR = 1.395, 95% CI: 1.033–1.883, *P* = 0.030). The genetic association power is 0.984 for rs2071746T/A SNP. No statistically significant differences were observed between the two groups for the (GT)n repeat polymorphism of *HMOX1* when analyzed using different genetic models (*P* > 0.05; **Table 2**).

The combined genotypes of *HMOX1* rs2071746T/A and (GT)n polymorphisms exhibited a significant difference in frequency between patients with PCOS and controls (*P* = 0.031; **Supplementary Table 1**). The TT/SS was a risk factor for PCOS (OR = 1.442, 95% CI: 1.021–2.035, *P* = 0.037) in a multinomial logistic regression model using the AA/LL combined genotype as the reference, with participant recruitment year, age, and BMI as covariates. Moderate linkage disequilibrium was observed between the rs2071746T/A and (GT)n polymorphisms (D’= 0.8517, r^2^ = 0.5539).

### 3.4 Effects of genotypes on clinical and biochemical indicators

We analyzed the effect of *HMOX1* rs2071746T/A and (GT)n genetic variants on plasma HMOX1 levels and clinical and biochemical parameters in women with and without PCOS.

**Supplementary Table 2** shows that patients with the AT genotype of *HMOX1* rs2071746T/A SNP had a greater TOS/GSH ratio than those with the TT genotype (*P* = 0.028). The controls with the TT genotype exhibited a lower acne grade score than those with the AA genotype (*P* = 0.043) and lower HDL-C levels (*P* = 0.038) than those with the AT genotype; whereas the controls with the AT genotype exhibited lower GSH levels (*P* = 0.022) than those with the AA genotype.

The same parameters were analyzed for different genotypes of *HMOX1* (GT)n repeat polymorphism (**Supplementary Table 3**). Patients with the LL genotype displayed a lower waist-to-hip ratio and TT levels (*P* < 0.05) than those with the SS genotype. The controls with the LL genotype exhibited a lower BMI than those with the SS and SL genotypes (*P* < 0.05). The controls with the SL genotype showed higher FAI (*P* = 0.019) than those with the SS genotype.

No statistically significant differences in plasma HMOX1 levels were observed between the different genotypes of *HMOX1* rs2071746T/A and (GT)n genetic variants in the control and PCOS groups (*P* > 0.05; **Supplementary Tables 2**, **3**).

## 4 Discussion

For the first time, we demonstrated that the TT genotype and T allele of the rs2071746T/A SNP are associated with an increased risk of PCOS in Chinese women. We also proved that the TT/SS combined genotype of the rs2071746T/A and (GT)n repeat variants is a risk factor for PCOS. Furthermore, we found that plasma HMOX1 levels were significantly higher in patients with PCOS than those in the control women, and elevated HMOX1 levels were related to a slight but significant increase in the risk of PCOS, suggesting that patients with PCOS have a compensatory increase in HMOX1 levels. *HMOX1* rs2071746T/A and (GT)n repeat polymorphisms significantly affected BMI, waist-to-hip ratio, TT, FAI, acne grade score, HDL-C, GSH, and TOS/GSH ratio, but not plasma HMOX1 levels among the PCOS and/or control participants, supporting that the two variants may be involved in obesity, endocrine abnormalities, oxidative stress, and metabolic disorders.

Oxidative stress, metabolic disorders, and iron homeostasis imbalance play significant roles in the occurrence and progression of PCOS (18–21, 32). HMOX catalyzes the degradation of heme and is crucial for controlling the dynamic equilibrium of heme and its products (BV, BR, CO, and Fe^2+^) (7, 8). In addition to the recovery of Fe^2+^ from heme, HMOX participates in the regulation of multiple signaling pathways via its products, BV, CO, and Fe^2+^, as well as its substrate, heme (8). Under physiological conditions, low HMOX1 expression is found in most tissues, except in cells of the reticuloendothelial system (8, 10). Unlike the constitutive isoform HMOX2, which is barely regulated at the transcriptional and translational levels (5, 8), HMOX1 can be rapidly induced under various stress conditions (7, 8, 10, 11). BV and BR are important endogenous antioxidants and cellular signaling molecules that play significant roles in regulating immunity and glycolipid metabolism, and CO is a gaseous mediator with vasodilatory, anti-inflammatory, anti-proliferative, and anti-apoptotic properties, while heme and free Fe^2+^ can facilitate the production of reactive oxygen species (7, 8, 10). Therefore, besides its cytoprotective effects, HMOX1 induction may also be involved in the development of certain diseases. Several studies have indicated that genetic overexpression or chemical induction of HMOX1 can protect against hypertension, cardiovascular diseases, metabolic conditions, and kidney diseases (33–35). Increased HMOX1 activity may promote oxidative stress by increasing free intracellular iron and accelerating the consumption of cytosolic NADPH, thereby contributing to chronic inflammation, ferroptosis, and cell injury (8). It has been reported that elevated plasma HMOX1 levels in individuals with T2D are associated with a higher disease risk (36). However, another study showed that low serum HMOX1 levels in non-obese women are an independent risk factor for PCOS (24). In this study, we found that plasma HMOX1 levels were significantly higher in the PCOS group compared to the control group, and the lean PCOS subgroup compared to the lean control subgroup. Furthermore, elevated HMOX1 levels were related to an increased risk of PCOS. The possible reasons for the inconsistent results of HMOX1 levels in PCOS may be discrepancies in the sample size and study population. Contrary to the report of lower HMOX1 concentrations in PCOS (24), our finding of elevated HMOX1 levels could represent a protective compensatory response to various chronic unfavorable stimuli in PCOS, aligning with the canonical role of HMOX1 as an oxidative stress sensor.

Increased oxidative stress in PCOS, as shown in this study, can enhance *HMOX1* transcription by activating nuclear factor erythroid 2-related factor 2 transcription factor (11, 37). Patients with PCOS have iron overload, abnormal heme metabolism, and chronic inflammation due to chronic oligomenorrhea, excessive androgen, and compensatory hyperinsulinemia (32, 38). High levels of heme and activation of the inflammatory factor nuclear factor kappa B can promote the expression of *HMOX1* (11, 33). The HMOX1 and its downstream metabolites, including CO, BV, and BR, may play a protective role through their antioxidant, anti-inflammatory, and vasodilator functions (8, 10). However, the sustained induction of *HMOX1* and the iron overload may paradoxically dysregulate ferroptosis through iron-mediated production of peroxidized lipids, potentially contributing to ovarian dysfunction. In addition to transcriptional regulation, HMOX1 activity is regulated by critical protein-protein interactions (PPIs) and post-translational modifications (39). PPIs affect the stability, oligomerization, subcellular localization, and function of HMOX1 (39–42). The post-translational modifications, such as phosphorylation, acetylation, and ubiquitination, also play an important role in regulating the level and activity of HMOX1 (43–45). In a word, the possible reasons and mechanisms for elevated HMOX1 levels are complex in PCOS. Further studies are needed to explain this phenomenon and its exact pathophysiological mechanisms.

Genetic variants in the promoter of *HMOX1* may affect the expression of *HMOX1* (11), thereby affecting the incidence and progression of diseases. The A allele of *HMOX1* rs2071746T/A SNP is associated with a higher transcription activity of *HMOX1* (11, 46). A meta-analysis revealed a lower susceptibility to coronary heart disease in individuals carrying the A allele (11). The AA genotype increases the occurrence of hypertension in the Japanese women (47), but decreases the risk of ischemic heart disease in the Japanese population (48). The T allele of the rs2071746T/A variant is a risk factor for the development of esophageal varices in patients with cirrhosis (46), and the TT genotype is more likely to cause proteinuria in Korean patients with T2D (49) and SARS-CoV-2 viremia in COVID-19 infection (16). Our findings indicated that women with the TT genotype and T allele have a higher risk of developing PCOS. Moreover, we found that this genetic polymorphism may contribute to oxidative stress, hyperandrogenism status, and metabolic disorders through influencing TOS/GSH ratio, GSH and HDL-C levels, and acne grade score in the study population. However, we did not observe significant differences in plasma HMOX1 levels according to different genotypes of the rs2071746T/A SNP, suggesting that this genetic variant may not be a key factor affecting HMOX1 expression in the study population.

Of the polymorphisms observed in the *HMOX1* promoter region, the (GT)n repeat variant has been extensively studied (11, 13). The range of (GT)n repeat numbers is 10–50 (17), and the number of repeat lengths shows a bimodal distribution, with peaks at (GT)_23_ and (GT)_30_ repeats in East Asian and Caucasian populations, and a trimodal form, with crest values at (GT)_23_, (GT)_30_, and (GT)_39_ among African-Americans (11). Generally, the short (S) allele is defined as the number of (GT)n repeats < 25 or 27, and the long (L) allele is defined as the number of (GT)n repeats ≥ 25 or 27 in different reports (11–13, 49). The S alleles are linked to increased transcriptional activity compared with the L alleles (11, 13), and individuals with the SS genotype of the (GT)n variant have higher levels of *HMOX1* mRNA than those with the LL genotype (50). Individuals with the S allele or SS genotype have a reduced risk of coronary heart disease (11), T2D (13), rheumatoid arthritis (50), and encephalitis in HIV infection (17), but an increased risk of melanoma (51). Whereas individuals with the L allele or LL genotype have an increased risk of hypertension (52), chronic obstructive pulmonary disease (15), and preeclampsia (late-onset and non-severe form) (14). Our study revealed no significant differences were observed between the PCOS and control groups based on different genetic models. However, this polymorphism may be involved in obesity and endocrine disorders, probably by affecting waist-to-hip ratio, BMI, TT levels, and FAI in the study population. Moreover, we did not observe significant differences in plasma HMOX1 levels according to different genotypes of the (GT)n repeat variant.

Additionally, in this study, a moderate linkage disequilibrium was observed between the rs2071746T/A and (GT)n repeat polymorphisms. The TT/SS combined genotype of the two genetic polymorphisms was a risk factor for PCOS. However, we did not observe significant differences in the HMOX1 levels according to the different combined genotypes in the control and PCOS groups (*P* > 0.05, data not indicated). Several studies have shown that *HMOX1* expression is also controlled by genetic polymorphisms (11). The (GT)n and rs2071746T/A polymorphisms may be involved in 5’-UTR alternative splicing of the *HMOX1* primary transcript, which may affect translational efficiency and mRNA stability, and thus regulate the translational process of *HMOX1* (53). Therefore, a more detailed and systematic investigation of the correlation between genotype and gene expression, along with *in vitro* studies, is required to clarify the potential mechanism.

The present study has some limitations. First, we did not measure the levels of BV, BR, and iron, which are critical downstream products of HMOX1 enzymatic activity; this could provide further evidence to reveal the relationships between *HMOX1* genetic variants, HMOX1 levels, and PCOS and the underlying mechanism. Second, we did not evaluate oxidative stress and hormonal and metabolic indices because of confounding factors in some participants, which could have had an effect on the statistical effectiveness of these parameters. Third, lifestyle factors (e.g., dietary patterns, physical activity levels) and longitudinal treatment histories were not collected during enrollment, preventing us from adjusting for differences in these potential confounders in our analyses.

In conclusion, this study indicates that the *HMOX1* rs2071746T/A SNP is associated with the risk of PCOS, and that the T allele, TT genotype, and its coexistence with the SS genotype of the (GT)n repeat variant are genetic risk factors for PCOS among Chinese women. We further demonstrated that patients with PCOS have higher plasma HMOX1 concentrations and that elevated HMOX1 levels are associated with an increased risk of PCOS. We found that the *HMOX1* rs2071746T/A and (GT)n repeat polymorphisms may contribute to obesity, oxidative stress, endocrine abnormalities, and metabolic disorders. Our findings suggest that induction of the heme-degrading enzyme HMOX1 and its genetic polymorphisms in the promoter may be involved in the pathophysiology of PCOS.

## Data Availability

The original contributions presented in the study are included in the article/**Supplementary Material**. Further inquiries can be directed to the corresponding authors.

## References

[1] Teede HJ Tay CT Laven J Dokras A Moran LJ Piltonen TT . Recommendations from the 2023 international evidence-based guideline for the assessment and management of polycystic ovary syndrome. Fertil Steril. (2023) 120:767–93. doi: 10.1016/j.fertnstert.2023.07.025, PMID: 37589624

[2] Escobar-Morreale HF . Polycystic ovary syndrome: definition, aetiology, diagnosis and treatment. Nat Rev Endocrinol. (2018) 14:270–84. doi: 10.1038/nrendo.2018.24, PMID: 29569621

[3] Joshi A . PCOS stratification for precision diagnostics and treatment. Front Cell Dev Biol. (2024) 12:1358755. doi: 10.3389/fcell.2024.1358755, PMID: 38389707 PMC10881805

[4] Palumbo M Della Corte L Colacurci D Ascione M D'Angelo G Baldini GM . PCOS and the genome: is the genetic puzzle still worth solving? Biomedicines. (2025) 13:1912. doi: 10.3390/biomedicines13081912, PMID: 40868166 PMC12383298

[5] Zhang X Li S Liu H Bai H Liu Q Yang C . Heme oxygenase 2 genetic variants alter hormonal and metabolic traits in polycystic ovary syndrome. Endocr Connect. (2024) 13:e230463. doi: 10.1530/EC-23-0463, PMID: 38251965 PMC10895317

[6] Merkin SS Phy JL Sites CK . Yang D Environmental determinants of polycystic ovary syndrome. Fertil Steril. (2016) 106:16–24. doi: 10.1016/j.fertnstert.2016.05.011, PMID: 27240194

[7] Haines DD . Tosaki A heme degradation in pathophysiology of and countermeasures to inflammation-associated disease. Int J Mol Sci. (2020) 21:9698. doi: 10.3390/ijms21249698, PMID: 33353225 PMC7766613

[8] Duvigneau JC Esterbauer H . Kozlov AV role of heme oxygenase as a modulator of heme-mediated pathways. Antioxidants (Basel). (2019) 8:475. doi: 10.3390/antiox8100475, PMID: 31614577 PMC6827082

[9] Kim YM Pae HO Park JE Lee YC Woo JM Kim NH . Heme oxygenase in the regulation of vascular biology: from molecular mechanisms to therapeutic opportunities. Antioxid Redox Signal. (2011) 14:137–67. doi: 10.1089/ars.2010.3153, PMID: 20624029 PMC2988629

[10] Nitti M Furfaro AL . Mann GE heme oxygenase dependent bilirubin generation in vascular cells: A role in preventing endothelial dysfunction in local tissue microenvironment? Front Physiol. (2020) 11:23. doi: 10.3389/fphys.2020.00023, PMID: 32082188 PMC7000760

[11] Ma LL Sun L Wang YX Sun BH Li YF . Jin YL Association between HO−1 gene promoter polymorphisms and diseases (Review). Mol Med Rep. (2022) 25:29. doi: 10.3892/mmr.2021.12545, PMID: 34841438 PMC8669660

[12] Chen YH Hung SC . Tarng DC Length polymorphism in heme oxygenase-1 and cardiovascular events and mortality in hemodialysis patients. Clin J Am Soc Nephrol. (2013) 8:1756–63. doi: 10.2215/CJN.01110113, PMID: 23813560 PMC3789334

[13] Rivera-Valdes JJ Sifuentes-Franco S Ramirez-Meza SM Iniguez-Mosqueda O Morales-Nunez JJ Ramirez-Evangelista ML . Association between microsatellite polymorphism in the Heme Oxygenase-1 (HMOX1) gene promoter and type 2 diabetes: an updated meta-analysis. J Diabetes Metab Disord. (2025) 24:63. doi: 10.1007/s40200-025-01575-y, PMID: 39917725 PMC11794720

[14] Kaartokallio T Klemetti MM Timonen A Uotila J Heinonen S Kajantie E . Microsatellite polymorphism in the heme oxygenase-1 promoter is associated with nonsevere and late-onset preeclampsia. Hypertension. (2014) 64:172–7. doi: 10.1161/HYPERTENSIONAHA.114.03337, PMID: 24799610

[15] Zhou H Ying X Liu Y Ye S Yan J . Li Y Genetic polymorphism of heme oxygenase 1 promoter in the occurrence and severity of chronic obstructive pulmonary disease: a meta-analysis. J Cell Mol Med. (2017) 21:894–903. doi: 10.1111/jcmm.13028, PMID: 27998018 PMC5387120

[16] Roy-Vallejo E Fernandez De Cordoba-Onate S Delgado-Wicke P Triguero-Martinez A Montes N Carracedo-Rodriguez R . Occurrence of SARS-CoV-2 viremia is associated with genetic variants of genes related to COVID-19 pathogenesis. Front Med (Lausanne). (2023) 10:1215246. doi: 10.3389/fmed.2023.1215246, PMID: 37809329 PMC10557488

[17] Gill AJ Garza R Ambegaokar SS Gelman BB Kolson DL . Heme oxygenase-1 promoter region (GT)n polymorphism associates with increased neuroimmune activation and risk for encephalitis in HIV infection. J Neuroinflammation. (2018) 15:70. doi: 10.1186/s12974-018-1102-z, PMID: 29510721 PMC5838989

[18] Zhang R Hu K Bai H Liu H Pu Y Yang C . Increased oxidative stress is associated with hyperandrogenemia in polycystic ovary syndrome evidenced by oxidized lipoproteins stimulating rat ovarian androgen synthesis *in vitro*. Endocrine. (2024) 84:1238–49. doi: 10.1007/s12020-024-03726-2, PMID: 38374513

[19] Armanini D Boscaro M Bordin L Sabbadin C . Controversies in the pathogenesis, diagnosis and treatment of PCOS: focus on insulin resistance, inflammation, and hyperandrogenism. Int J Mol Sci. (2022) 23:4110. doi: 10.3390/ijms23084110, PMID: 35456928 PMC9030414

[20] Sun Y Li S Liu H Bai H Hu K Zhang R . Oxidative stress promotes hyperandrogenism by reducing sex hormone-binding globulin in polycystic ovary syndrome. Fertil Steril. (2021) 116:1641–50. doi: 10.1016/j.fertnstert.2021.07.1203, PMID: 34433519

[21] Zhang R Liu H Bai H Zhang Y Liu Q Guan L . Oxidative stress status in Chinese women with different clinical phenotypes of polycystic ovary syndrome. Clin Endocrinol (Oxf). (2017) 86:88–96. doi: 10.1111/cen.13171, PMID: 27489079

[22] Liu S Zhao X Meng Q Li B . Screening of potential biomarkers for polycystic ovary syndrome and identification of expression and immune characteristics. PloS One. (2023) 18:e0293447. doi: 10.1371/journal.pone.0293447, PMID: 37883387 PMC10602247

[23] Manneras-Holm L Benrick A Stener-Victorin E . Gene expression in subcutaneous adipose tissue differs in women with polycystic ovary syndrome and controls matched pair-wise for age, body weight, and body mass index. Adipocyte. (2014) 3:190–6. doi: 10.4161/adip.28731, PMID: 25068085 PMC4110095

[24] Gao H Meng J Xing H Nie S Xu M Zhang S . Association of heme oxygenase-1 with the risk of polycystic ovary syndrome in non-obese women. Hum Reprod. (2014) 29:1058–66. doi: 10.1093/humrep/deu029, PMID: 24585089

[25] Rotterdam EA-SPCWG . Revised 2003 consensus on diagnostic criteria and long-term health risks related to polycystic ovary syndrome. Fertil Steril. (2004) 81:19–25. doi: 10.1016/j.fertnstert.2003.10.004, PMID: 14711538

[26] Liu Q Liu H Bai H Huang W Zhang R Tan J . Association of SOD2 A16V and PON2 S311C polymorphisms with polycystic ovary syndrome in Chinese women. J Endocrinol Invest. (2019) 42:909–21. doi: 10.1007/s40618-018-0999-5, PMID: 30607774

[27] Zhang J Zhang Y Liu H Bai H Wang Y Jiang C . Antioxidant properties of high-density lipoproteins are impaired in women with polycystic ovary syndrome. Fertil Steril. (2015) 103:1346–54. doi: 10.1016/j.fertnstert.2015.02.024, PMID: 25813288

[28] Zhang J Fan P Liu H Bai H Wang Y Zhang F Apolipoprotein A-I . and B levels, dyslipidemia and metabolic syndrome in south-west Chinese women with PCOS. Hum Reprod. (2012) 27:2484–93. doi: 10.1093/humrep/des191, PMID: 22674204

[29] Robert Y Dubrulle F Gaillandre L Ardaens Y Thomas-Desrousseaux P Lemaitre L . Ultrasound assessment of ovarian stroma hypertrophy in hyperandrogenism and ovulation disorders: visual analysis versus computerized quantification. Fertil Steril. (1995) 64:307–12. doi: 10.1016/S0015-0282(16)57728-0, PMID: 7615108

[30] Higuchi R . PCR technology. In: Erlich HA , editor. Principles and applications for DNA amplification, 1st edn. Stockton Press, New York (1989). p. 36.

[31] Seu L Burt TD Witte JS Martin JN Deeks SG McCune JM . Variations in the heme oxygenase-1 microsatellite polymorphism are associated with plasma CD14 and viral load in HIV-infected African-Americans. Genes Immun. (2012) 13:258–67. doi: 10.1038/gene.2011.76, PMID: 22048453 PMC3330188

[32] Escobar-Morreale HF Luque-Ramirez M . Role of androgen-mediated enhancement of erythropoiesis in the increased body iron stores of patients with polycystic ovary syndrome. Fertil Steril. (2011) 95:1730–1735.e1731. doi: 10.1016/j.fertnstert.2011.01.038, PMID: 21300335

[33] Ryter SW . Heme oxygenase-1: an anti-inflammatory effector in cardiovascular, lung, and related metabolic disorders. Antioxidants (Basel). (2022) 11:555. doi: 10.3390/antiox11030555, PMID: 35326205 PMC8944973

[34] McClung JA Levy L Garcia V Stec DE Peterson SJ Abraham NG . Heme-oxygenase and lipid mediators in obesity and associated cardiometabolic diseases: Therapeutic implications. Pharmacol Ther. (2022) 231:107975. doi: 10.1016/j.pharmthera.2021.107975, PMID: 34499923 PMC8958338

[35] Raghunandan S Ramachandran S Ke E Miao Y Lal R Chen ZB . Heme oxygenase-1 at the nexus of endothelial cell fate decision under oxidative stress. Front Cell Dev Biol. (2021) 9:702974. doi: 10.3389/fcell.2021.702974, PMID: 34595164 PMC8476872

[36] Bao W Song F Li X Rong S Yang W Zhang M . Plasma heme oxygenase-1 concentration is elevated in individuals with type 2 diabetes mellitus. PloS One. (2010) 5:e12371. doi: 10.1371/journal.pone.0012371, PMID: 20811623 PMC2928270

[37] Kerins MJ Ooi A . The roles of NRF2 in modulating cellular iron homeostasis. Antioxid Redox Signal. (2018) 29:1756–73. doi: 10.1089/ars.2017.7176, PMID: 28793787 PMC6208163

[38] Luque-Ramirez M Alvarez-Blasco F Alpanes M Escobar-Morreale HF . Role of decreased circulating hepcidin concentrations in the iron excess of women with the polycystic ovary syndrome. J Clin Endocrinol Metab. (2011) 96:846–52. doi: 10.1210/jc.2010-2211, PMID: 21209031

[39] Jagadeesh ASV Fang X Kim SH Guillen-Quispe YN Zheng J Surh YJ . Non-canonical vs. Canonical functions of heme oxygenase-1 in cancer. J Cancer Prev. (2022) 27:7–15. doi: 10.15430/JCP.2022.27.1.7, PMID: 35419301 PMC8984652

[40] Song J Zhang X Liao Z Liang H Chu L Dong W . 14-3-3zeta inhibits heme oxygenase-1 (HO-1) degradation and promotes hepatocellular carcinoma proliferation: involvement of STAT3 signaling. J Exp Clin Cancer Res. (2019) 38:3. doi: 10.1186/s13046-018-1007-9, PMID: 30606233 PMC6319010

[41] Hwang HW Lee JR Chou KY Suen CS Hwang MJ Chen C . Oligomerization is crucial for the stability and function of heme oxygenase-1 in the endoplasmic reticulum. J Biol Chem. (2009) 284:22672–9. doi: 10.1074/jbc.M109.028001, PMID: 19556236 PMC2755675

[42] Lin Q Weis S Yang G Weng YH Helston R Rish K . Heme oxygenase-1 protein localizes to the nucleus and activates transcription factors important in oxidative stress. J Biol Chem. (2007) 282:20621–33. doi: 10.1074/jbc.M607954200, PMID: 17430897

[43] Hsu FF Chiang MT Li FA Yeh CT Lee WH Chau LY . Acetylation is essential for nuclear heme oxygenase-1-enhanced tumor growth and invasiveness. Oncogene. (2017) 36:6805–14. doi: 10.1038/onc.2017.294, PMID: 28846111

[44] Lin PH Lan WM Chau LY . TRC8 suppresses tumorigenesis through targeting heme oxygenase-1 for ubiquitination and degradation. Oncogene. (2013) 32:2325–34. doi: 10.1038/onc.2012.244, PMID: 22689053

[45] Salinas M Wang J Rosa de Sagarra M Martin D Rojo AI Martin-Perez J . Protein kinase Akt/PKB phosphorylates heme oxygenase-1 *in vitro* and in *vivo*. FEBS Lett. (2004) 578:90–4. doi: 10.1016/j.febslet.2004.10.077, PMID: 15581622

[46] Ellakany WI Mahmoud MoheyEldin K Invernizzi P Mahmoud ElKady A Eldin Fathy Abou Elkheir H Abdel Haleem Abo Elwafa R . Study of the influence of heme oxygenase 1 gene single nucleotide polymorphism (rs2071746) on esophageal varices among patients with cirrhosis. Eur J Gastroenterol Hepatol. (2018) 30:888–92. doi: 10.1097/MEG.0000000000001161, PMID: 29877949

[47] Ono K Mannami T Iwai N . Association of a promoter variant of the haeme oxygenase-1 gene with hypertension in women. J Hypertens. (2003) 21:1497–503. doi: 10.1097/00004872-200308000-00013, PMID: 12872043

[48] Ono K Goto Y Takagi S Baba S Tago N Nonogi H . A promoter variant of the heme oxygenase-1 gene may reduce the incidence of ischemic heart disease in Japanese. Atherosclerosis. (2004) 173:315–9. doi: 10.1016/j.atherosclerosis.2003.11.021, PMID: 15064108

[49] Lee EY Lee YH Kim SH Jung KS Kwon O Kim BS . Association between heme oxygenase-1 promoter polymorphisms and the development of albuminuria in type 2 diabetes: A case-control study. Med (Baltimore). (2015) 94:e1825. doi: 10.1097/MD.0000000000001825, PMID: 26512585 PMC4985399

[50] Rueda B Oliver J Robledo G Lopez-Nevot MA Balsa A Pascual-Salcedo D . HO-1 promoter polymorphism associated with rheumatoid arthritis. Arthritis Rheumatol. (2007) 56:3953–8. doi: 10.1002/art.23048, PMID: 18050210

[51] Okamoto I Krogler J Endler G Kaufmann S Mustafa S Exner M . A microsatellite polymorphism in the heme oxygenase-1 gene promoter is associated with risk for melanoma. Int J Cancer. (2006) 119:1312–5. doi: 10.1002/ijc.21937, PMID: 16596642

[52] Wu MM Chiou HY Chen CL Hsu LI Lien LM Wang CH . Association of heme oxygenase-1 GT-repeat polymorphism with blood pressure phenotypes and its relevance to future cardiovascular mortality risk: an observation based on arsenic-exposed individuals. Atherosclerosis. (2011) 219:704–8. doi: 10.1016/j.atherosclerosis.2011.08.047, PMID: 21945498

[53] Kramer M Sponholz C Slaba M Wissuwa B Claus RA Menzel U . Alternative 5' untranslated regions are involved in expression regulation of human heme oxygenase-1. PloS One. (2013) 8:e77224. doi: 10.1371/journal.pone.0077224, PMID: 24098580 PMC3788786

