# Correction: The promoter T-413A variant and elevated enzyme levels of heme oxygenase-1 associated with an increased risk of polycystic ovarian syndrome

**DOI:** 10.3389/fendo.2026.1739810

**Published:** 2026-01-20

**Authors:** Qiuyi Wang, Jiagui Liang, Qingqing Liu, Hongwei Liu, Huai Bai, Wei Huang, Ping Fan

**Affiliations:** 1 Department of Obstetrics and Gynecology, West China Second University Hospital, Sichuan University, Chengdu, Sichuan, China; 2 Laboratory of Genetic Disease and Perinatal Medicine, Key Laboratory of Birth Defects and Related Diseases of Women and Children, Ministry of Education, West China Second University Hospital, Sichuan University, Chengdu, Sichuan, China; 3 Department of Reproductive Medical Center, West China Second University Hospital, Sichuan University, Chengdu, Sichuan, China

**Keywords:** heme oxygenase-1, genetic polymorphism, polycystic ovarian syndrome, oxidative stress, metabolism

There were mistakes in **Table 2** as published:

- In the unadjusted OR (95% CI) column, the word ‘Referent’ missed in lines 3 and 18.
- Revision of data in the OR value in line 9 from 1.257 to 1.275 and the 95% CI value in last line from (0.950-0.239) to (0.950–1.229).
- Revision of data in the OR (95% CI) column, some hyphens from “-” to “–”].

The corrected **Table 2** appears below (The red font represents the modified contents).

**Table 2:** Association of *HMOX1* T-413A (rs2071746) and (GT)n repeat polymorphisms with the risk of PCOS using different genetic models.

Variants	Controls (n = 805)	PCOS (n = 1092)	Unadjusted		Adjusted	
			OR (95% CI)	*P*	OR (95% CI)	*P*
T-413A (rs2071746)						
Genotype						
AA	173 (21.5%)	193 (17.7%)	Referent			
AT	426 (52.9%)	556 (50.9%)	1.170 (0.919–1.489)	0.201	1.140 (0.872–1.493)	0.342
TT	206 (25.6%)	343 (31.4%)	1.493 (1.141–1.952)	0.003	1.395 (1.033–1.883)	0.030
*P*_HWE_	0.233	0.456				
Recessive						
TT + AT	632 (78.5%)	899 (82.3%)				
AA	173 (21.5%)	193 (17.7%)	1.275 (1.014–1.603)	0.037	1.212 (0.936–1.570)	0.144
Dominant						
TT	206 (25.6%)	343 (31.4%)				
AA + AT	599 (74.4%)	749 (68.6%)	1.332 (1.086–1.632)	0.006	1.272 (1.013–1.597)	0.039
Allele						
A	772 (48.0%)	942 (43.1%)				
T	838 (52.0%)	1242 (56.9%)	1.215 (1.067–1.382)	0.003	/	/
(GT)n repeat						
Genotype						
LL	224 (27.8%)	298 (27.3%)	Referent			
SL	422 (52.4%)	542 (49.6%)	0.965 (0.779–1.197)	0.748	0.935 (0.736–1.189)	0.584
SS	159 (19.8%)	252 (23.1%)	1.191 (0.915–1.551)	0.193	1.134 (0.844–1.522)	0.404
*P*_HWE_	0.292	0.983				
Recessive						
LL + SL	646 (80.2%)	840 (76.9%)				
SS	159 (19.8%)	252 (23.1%)	1.219 (0.975–1.524)	0.082	0.842 (0.655–1.081)	0.178
Dominant						
LL	224 (27.8%)	298 (27.3%)				
SS + SL	581 (72.2%)	794 (72.7%)	0.973 (0.794–1.193)	0.796	1.041 (0.827–1.309)	0.733
Allele						
L	870 (54.0%)	1138 (52.1%)				
S	740 (46.0%)	1046 (47.9%)	1.081 (0.950–1.229)	0.239	/	/

The original version of this article has been updated.

