# Supplementary material for: The promoter T-413A variant and elevated enzyme levels of heme oxygenase-1 associated with an increased risk of polycystic ovarian syndrome
Source: Front Endocrinol (Lausanne). 2025 Nov 4;16:1644373. doi: 10.3389/fendo.2025.1644373 (PMC12623185; doi:10.3389/fendo.2025.1644373)
Supplement: Supplementary file 1 [file Table1.docx]

**Supplement Table 1. Frequencies of combined genotypes of *HMOX1*** T-413A **(rs2071746) and (GT)n repeat in women with PCOS and controls**

| Genotype combinations | Controls  (n = 805) | PCOS  (n = 1092) | OR | 95%CI | *P* |
| --- | --- | --- | --- | --- | --- |
| T-413A and (GT)n repeat*** | | |  |  |  |
| AA/LL | 151 (18.8%) | 165 (15.1%) | 1.00 | - | - |
| AA/SL | 22 (2.7%) | 28 (2.6%) | 1.044 | 0.531–2.055 | 0.900 |
| AT/LL | 67 (8.3%) | 114 (10.4%) | 1.511 | 0.999–2.287 | 0.051 |
| AT/SL | 333 (41.4%) | 412 (37.7%) | 1.091 | 0.812-1.466 | 0.563 |
| AT/SS | 26 (3.2%) | 30 (2.7%) | 0.902 | 0.476-1.711 | 0.752 |
| TT/LL | 6 (0.7%) | 19 (1.7%) | 2.278 | 0.819-6.336 | 0.115 |
| TT/SL | 67 (8.3%) | 102 (9.3%) | 1.247 | 0.816-1.904 | 0.308 |
| TT/SS | 133 (16.5%) | 222 (20.3%) | 1.442 | 1.021–2.035 | 0.037 |

Data on genotype combinations are presented as number (%) of patients or controls.

*Comparisons of the frequencies between two groups using Chi-squared (χ²) analysis: *x^2^* = 15.462, *P* = 0.031. Odds ratio (OR) and 95% confidence interval (CI) were calculated using a multinomial logistic regression model including age, body mass index, and recruitment year of participants as covariates, and the AA/LL combined genotypes as the reference category.
