# Supplementary material for: The promoter T-413A variant and elevated enzyme levels of heme oxygenase-1 associated with an increased risk of polycystic ovarian syndrome
Source: Front Endocrinol (Lausanne). 2025 Nov 4;16:1644373. doi: 10.3389/fendo.2025.1644373 (PMC12623185; doi:10.3389/fendo.2025.1644373)
Supplement: Supplementary file 2 [file Table2.docx]

**Supplementary Table 2. Clinical and biochemical parameters according to *HMOX1* T-413A (rs2071746) genotypes in women with PCOS and controls**

|  | Controls | | |  | PCOS | | |
| --- | --- | --- | --- | --- | --- | --- | --- |
|  | AA  (n = 173) | AT  (n = 426) | TT  (n = 206) |  | AA  (n = 193) | AT  (n = 556) | TT  (n = 343) |
| Age (years) | 28.22 ± 4.29 | 28.42 ± 4.10 | 27.94 ± 3.96 |  | 24.90 ± 3.85 | 25.10 ± 4.14 | 25.22 ± 4.34 |
| BMI (kg/m^2^) | 20.88 ± 2.72 | 21.21 ± 2.79 | 21.25 ± 2.94 |  | 22.70 ± 3.78 | 23.11 ± 4.18 | 23.12 ± 4.26 |
| WC (cm) | 73.17 ± 8.13 | 73.88 ± 7.87 | 73.54 ± 8.49 |  | 78.30 ± 10.86 | 79.38 ± 11.35 | 79.24 ± 10.74 |
| Waist-to-hip ratio | 0.81 ± 0.06 | 0.82 ± 0.06 | 0.81 ± 0.06 |  | 0.85 ± 0.07 | 0.85 ± 0.07 | 0.85 ± 0.07 |
| F-G score | 0.27 ± 0.83 | 0.23 ± 0.67 | 0.24 ± 0.72 |  | 1.81 ± 2.07 | 1.70 ± 1.93 | 1.78 ± 2.15 |
| Acne grade score | 0.18 ± 0.39 | 0.13 ± 0.34 | 0.10 ± 0.30 ^a^ |  | 0.67 ± 0.91 | 0.66 ± 0.88 | 0.68 ± 0.92 |
| SBP (mmHg) | 112.00 ± 11.40 | 112.15 ± 10.94 | 113.17 ± 11.41 |  | 113.92 ± 9.85 | 114.45 ± 10.96 | 113.82 ± 10.48 |
| DBP (mmHg) | 73.68 ± 9.81 | 73.10 ± 7.88 | 73.54 ± 8.94 |  | 75.88 ± 9.02 | 75.28 ± 8.48 | 75.41 ± 9.05 |
| Mean ovarian volume (mL) | 7.00 ± 2.75 | 7.43 ± 2.70 | 7.47 ± 2.69 |  | 9.89 ± 4.51 | 9.92 ± 3.87 | 9.93 ± 4.09 |
| **Hormonal levels*** |  |  |  |  |  |  |  |
| E_2_ (pmol/L) | 317.85 ± 286.32 | 317.48 ± 307.71 | 303.45 ± 383.39 |  | 270.79 ± 234.41 | 275.83 ± 258.34 | 276.75 ± 317.51 |
| TT (nmol/L) | 1.38 ± 0.52 | 1.48 ± 0.51 | 1.44 ± 0.54 |  | 2.21 ± 0.74 | 2.26 ± 0.81 | 2.30 ± 0.81 |
| SHBG (nmol/L) | 54.31 ± 25.26 | 54.98 ± 27.23 | 56.84 ± 28.91 |  | 33.44 ± 19.34 | 32.39 ± 18.73 | 32.61 ± 19.31 |
| FAI | 3.15 ± 2.30 | 3.27 ± 1.91 | 2.86 ± 1.45 |  | 9.00 ± 6.50 | 9.66 ± 7.10 | 9.57 ± 6.68 |
| LH (IU/L) | 7.08 ± 5.79 | 7.18 ± 6.25 | 6.79 ± 6.61 |  | 12.80 ± 8.52 | 13.25 ± 7.82 | 13.18 ± 8.15 |
| FSH (IU/L) | 6.58 ± 2.58 | 6.58 ± 2.77 | 6.44 ± 2.39 |  | 5.81 ± 1.86 | 6.15 ± 2.24 | 6.02 ± 1.97 |
| LH/FSH | 1.14 ± 0.84 | 1.19 ± 1.14 | 1.12 ± 1.32 |  | 2.24 ± 1.20 | 2.24± 1.26 | 2.23 ± 1.20 |
| **Metabolic profile*** |  |  |  |  |  |  |  |
| Fasting Ins (pmol/L) | 59.42 ± 38.20 | 60.12 ± 33.24 | 61.71 ± 37.59 |  | 94.44 ± 68.47 | 98.49 ± 71.76 | 99.58 ± 75.90 |
| 2-h Ins (pmol/L) | 351.09 ± 283.82 | 381.77 ± 255.18 | 344.91 ± 292.45 |  | 718.72 ± 575.72 | 699.97 ± 540.63 | 737.85 ± 605.60 |
| Fasting Glu (mmol/L) | 5.23 ± 0.44 | 5.25 ± 0.52 | 5.18 ± 0.40 |  | 5.28 ± 1.06 | 5.36 ± 0.74 | 5.35 ± 0.85 |
| 2-h Glu (mmol/L) | 5.76 ± 1.23 | 6.12 ± 1.36 | 5.88 ± 1.10 |  | 6.99 ± 1.71 | 7.25 ± 2.48 | 7.28 ± 2.78 |
| HOMA-IR | 2.21 ± 1.46 | 2.22 ± 1.21 | 2.17 ± 1.32 |  | 3.57 ± 2.43 | 3.87 ± 3.09 | 3.81 ± 3.18 |
| TG (mmol/L) | 1.01 ± 0.67  ± | 0.98 ± 0.50 | 1.03 ± 0.47 |  | 1.24 ± 0.69 | 1.45± 1.26 | 1.44 ± 1.12 |
| TC (mmol/L) | 4.27 ± 0.64 | 4.27 ± 0.74 | 4.19 ± 0.74 |  | 4.44 ± 0.83 | 4.44 ± 0.80 | 4.40 ± 0.79 |
| HDL-C (mmol/L) | 1.53 ±0.29 | 1.54 ± 0.34 | 1.46 ± 0.31 ^b^ |  | 1.42 ± 0.34 | 1.37 ± 0.35 | 1.35 ± 0.32 |
| LDL-C (mmol/L) | 2.41 ± 0.61 | 2.36 ± 0.63 | 2.35 ± 0.68 |  | 2.61 ± 0.77 | 2.60 ± 0.78 | 2.59 ± 0.76 |
| TG/HDL-C | 0.73 ± 0.69 | 0.72 ± 0.64 | 0.76 ± 0.47 |  | 0.99 ± 0.74 | 1.24 ± 1.39 | 1.24 ± 1.58 |
| ApoA1 (g/L) | 1.48 ± 0.19 | 1.47 ± 0.22 | 1.44 ± 0.20 |  | 1.44 ± 0.22 | 1.41 ± 0.20 | 1.43 ± 0.21 |
| ApoB (g/L) | 0.75 ± 0.16 | 0.75 ± 0.17 | 0.74 ± 0.18 |  | 0.82 ± 0.21 | 0.83 ± 0.19 | 0.82 ± 0.20 |
| **Oxidative stress parameters*** | | |  |  |  |  |  |
| TOS (nmol H_2_O_2_ Equiv./mL) | 11.73 ± 6.00 | 11.28 ± 5.29 | 11.42 ± 4.88 |  | 14.86 ± 10.37 | 15.68 ± 10.39 | 14.75 ± 10.18 |
| T-AOC (U/mL/min) | 14.36 ± 2.56 | 14.55 ± 2.64 | 14.54 ± 2.57 |  | 15.77 ± 3.45 | 16.05 ± 3.69 | 15.88 ± 3.25 |
| OSI | 0.84 ± 0.50 | 0.77 ± 0.38 | 0.80 ± 0.35 |  | 0.99 ± 0.76 | 1.03 ± 0.84 | 0.92 ± 0.62 |
| GSH (nmol/mL) | 1.15 ± 0.29 | 1.08 ± 0.22 ^a^ | 1.13 ± 0.25 |  | 1.16 ± 0.23 | 1.18 ± 0.24 | 1.20 ± 0.28 |
| TOS/GSH | 10.34 ± 6.02 | 10.61 ± 5.91 | 10.41 ± 5.25 |  | 12.92 ± 10.78 | 13.48 ± 10.20 | 11.37 ± 6.60 ^c^ |
| HMOX1 (μg/L) | 4.48 ± 2.44 | 4.55 ± 2.42 | 4.36 ± 1.97 |  | 4.65 ± 3.25 | 5.11 ± 4.54 | 5.77 ± 5.35 |

Values are presented as average ± standard deviation.

apoA1, apolipoprotein A1; apoB, apolipoprotein B; BMI, body mass index; DBP, diastolic blood pressure; E_2_, estradiol; FAI, free androgen index; F-G score, Ferriman–Gallwey score; FSH, follicle-stimulating hormone; Glu, glucose; GSH, glutathione; HDL-C, high-density lipoprotein cholesterol; HMOX1, heme oxygenase-1; HOMA-IR, the homeostatic model assessment of insulin resistance; Ins, insulin; LDL-C, low-density lipoprotein cholesterol; LH, luteinizing hormone; OSI, oxidative stress index; SBP, systolic blood pressure; SHBG, sex hormone-binding globulin; T-AOC, total antioxidant capacity; TC, total cholesterol; TG, triglycerides; TOS, total oxidant status; TT, total testosterone; WC, waist circumference. 2-h Ins and 2-h Glu, 2-hour plasma insulin and glucose after the glucose challenge.

Comparisons of all parameters between the two subgroups were corrected for differences in age, BMI, and recruitment year of participants using analysis of covariance, except for age and BMI (using one-way analysis of variance).

^a^*P* < 0.05, compared with the AA genotype subgroup in the control group; ^b^*P* < 0.05, compared with the AT genotype subgroup in the control group; ^c^*P* < 0.05, compared with the AT genotype subgroup in the PCOS group.

*Controls (AA = 113, AT = 269, AA = 134); PCOS (AA = 131, AT = 373, TT = 222)
